# On the existence of positive solutions for fractional differential inclusions at resonance

**DOI:** 10.1186/s40064-016-2665-8

**Published:** 2016-07-02

**Authors:** Lei Hu

**Affiliations:** School of Science, Shandong Jiaotong University, Jinan, 250357 China

**Keywords:** Fractional differential inclusions, Multi-valued operator, Positive solution, Resonance, 34A60, 34A08, 34B15

## Abstract

In this paper, we discuss the existence of positive solutions for a boundary value problem of fractional differential inclusions with resonant boundary conditions. By using the Leggett–Williams theorem for coincidences of multi-valued operators due to O’Regan and Zima, results on the existence of positive solutions are established. An example is given to illustrate the efficiency of the main theorems.

## Background

In this article, we investigate the existence of positive solutions of fractional differential inclusions with two-point boundary conditions:

1 $${\left\{ \begin{array}{ll} D_{0^+}^{\alpha }u(t)\in f\left( t,u(t)\right) ,\quad 0\le t\le 1, \\ u^{(i)}(0)=0, ~u(0)=u(1),\quad i=1,2,\ldots ,n-1, \end{array}\right. }$$

where $n-1<\alpha <n , n\ge 2$, $D_{0^+}^{\alpha }$ denotes the Caputo fractional derivative, $f:[0,1]\times \mathbb {R}\rightarrow \mathcal {F}(\mathbb {R})$, $\mathcal {F}(\mathbb {R})$ denotes the family of nonempty compact and convex subsets of $\mathbb {R}$.

Fractional calculus is a generalization of ordinary differentiation and integration to arbitrary order. The fractional differential equations play an important role in various fields of science and engineering, such as chemistry, biology, control theory, viscoelastic materials, signal processing, finance, life science and so on, see Kilbas et al. (2006), Samko et al. (1993), Podlubny (1999) and Orsingher and Beghin (2004).

During the last 10 years, boundary value problems for fractional differential equations are one of the most active fields in the researches of nonlinear differential equations theories. For further details, see Bai and Lü (2005), Zhang (2006), Caballero et al. (2011), Xu et al. (2009), Lin (2007) and Goodrich (2010). Meanwhile, fractional boundary value problems at resonance have been extensively studied. For some recent works on the topic, see Kosmatov (2008, 2010), Bai (2011), Bai and Zhang (2011) and Yang and Wang (2011) and references therein. It is well known that differential inclusions have proved to be valuable tools in the modeling of many realistic problems, such as economics, optimal control and so on. Recently, fractional differential inclusions have been investigated by several researchers, we refer the reader to Agarwal et al. (2010) and Chen et al. (2013).

As shown in the above mentioned works, we can see two facts. Firstly, although the boundary value problems for fractional differential equations at resonance have been studied by some authors, the existence of positive solutions to fractional differential equations at resonance are seldom considered. Secondly, there are few papers to deal with fractional differential inclusions under resonant conditions. The study of positive solutions for higher-order fractional differential inclusions under resonant conditions has yet to be initiated.

To fill this gap, we discuss the fractional differential inclusions (1) by using the Leggett–Williams theorem for coincidences of multi-valued operators due to O’Regan and Zima (2008).

The rest of this paper is organized as follows. “Preliminaries” section, we give some necessary notations, definitions and lemmas. In “Main results” section, we obtain the existence of positive solutions of (1) by Theorem 1. Finally, an example is given to illustrate our results in “Example” section.

## Preliminaries

First of all, we present the necessary definitions and lemmas from fractional calculus theory. For more details, see Kilbas et al. (2006), Samko et al. (1993) and Podlubny (1999).

### **Definition 1**

(*Kilbas et al.*^2006^) The Riemann–Liouville fractional integral of order $\alpha >0$ of a function $f:(0,\infty )\rightarrow \mathbb {R}$ is given by

$$I_{0+}^{\alpha }f(t)=\frac{1}{\Gamma (\alpha )}\int _0^t(t-s)^{\alpha -1}f(s)ds,$$

provided that the right-hand side is pointwise defined on $(0,\infty )$.

### **Definition 2**

(*Kilbas et al.*^2006^) The Caputo fractional derivative of order $\alpha >0$ of a continuous function $f:(0,\infty )\rightarrow \mathbb {R}$ is given by

$$D_{0^+}^{\alpha }f(t)=\frac{1}{\Gamma (n-\alpha )}\int _{0}^{t}(t-s)^{n-\alpha -1}f^{(n)}(s)ds,$$

where $n-1<\alpha \le n$, provided that the right-hand side is pointwise defined on $(0,\infty )$.

### **Lemma 1**

(Kilbas et al. 2006) *The fractional differential equation*

$$D_{0+}^{\alpha }y(t)=0$$

*has solution*$$y(t)=c_{0}+c_{1}t+\cdots +c_{n-1}t^{n-1}, c_{i}\in \mathbb {R}$$*,*$$i=0,1,\ldots , n-1$$*,*$$n=[\alpha ]+1$$.

*Furthermore, for*$$y\in AC^n[0,1]$$*,*

$$\left( I_{0+}^{\alpha }D_{0+}^{\alpha }y\right) (t)=y(t)-\sum _{k=0}^{n-1}\frac{y^{(k)}(0)}{k!}t^k$$

*and*

$$\left( D_{0+}^{\alpha }I_{0+}^{\alpha }y\right) (t)=y(t).$$

### **Lemma 2**

(Kilbas et al. 2006) *The relation*

$$I_{a+}^{\alpha }I_{a+}^{\beta }f(x)=I_{a+}^{\alpha +\beta }f(x),$$

*is valid in following case:*$\beta>0,~\alpha +\beta >0,~f\in L^{1}(a,b).$

In the following, let us recall some definitions on Fredholm operators and cones in Banach space (see Mawhin 1979).

Let *X*, *Y* be real Banach spaces. Consider a linear mapping $L:{\text {dom}}L\subset X\rightarrow Y$ and a nonlinear multivalued mapping $N:X\rightarrow 2^Y$. Assume that

1. *L* is a Fredholm operator of index zero, that is, ${\text {Im}}L$ is closed and ${\text {dim (Ker}} L)={\text {codim(Im}}L)<\infty$,
2. $N:X\rightarrow 2^Y$ is an upper semicontinuous mapping with nonempty compact convex values.

The assumption (A1) implies that there exist continuous projections $P:X\rightarrow X$ and $Q:Y\rightarrow Y$ such that ${\text {Im}}P=\text {Ker} L$ and $\text {Ker} Q={\text {Im}}L$. Moreover, since $\text {dim (Im}Q)={\text {codim (Im}}L)$, there exists an isomorphism $J:{\text {Im}}Q\rightarrow \text {Ker} L$. Denote by $L_p$ the restriction of *L* to $\text {Ker} P\cap {\text {dom}}L$. Clearly, $L_p$ is an isomorphism from $\text {Ker} P\cap {\text {dom}}L$ to ${\text {Im}}L$, we denote its inverse by $K_p:{\text {Im}}L\rightarrow \text {Ker} P\cap {\text {dom}}L$. It is known that the inclusion $Lx\in Nx$ is equivalent to

$$x\in \left( P+JQN\right) x+K_P(I-Q)Nx.$$

Let *C* be a cone in *X* such that

1. $\mu x\in C$ for all $x\in C$ and $\mu \ge 0$,
2. $$x,-x\in C$$ implies $$x=\theta$$.

It is well known that *C* induces a partial order in *X* by

$$x\preceq y \quad \text {if and only if}\, y-x\in C.$$

The following property is valid for every cone in a Banach space *X*.

### **Lemma 3**

*Let C be a cone in X. Then for every*$u\in C{\setminus} \{0\}$*there exists a positive number*$\sigma (u)$*such that*

$$\Vert x+u\Vert \ge \sigma (u)\Vert u\Vert \quad \text {for all }x\in C.$$

Let $\gamma :X\rightarrow C$ be a retraction, that is, a continuous mapping such that $\gamma (x)=x$ for all $x\in C$. Set

$$\Psi :=P+JQN+K_p(I-Q)N\quad \text {and}\quad \Psi _\gamma :=\Psi \circ \gamma.$$

We use the following result due to O’Regan and Zima.

### **Theorem 1**

(O’Regan and Zima 2008) *Let C be a cone in X and let*$\Omega _1$*,*$\Omega _2$*be open bounded subsets of X with*$\overline{\Omega }_1\subset \Omega _2$*and*$C\cap (\overline{\Omega }_2{\setminus }\Omega _1)\ne \emptyset$*. Assume that (A1), (A2) hold and the following assumptions hold:*

(A3) $QN:X\rightarrow 2^Y$*is bounded on bounded subsets ofC and *$K_p(I-Q)N:X\rightarrow 2^X$*be compact on every bounded subset of C,*
(A4) $\gamma$*maps subsets of*$\overline{\Omega }_2$*into bounded subsets of C,*
(A5) $Lx\notin \lambda Nx$*for all*$x\in C\cap \partial \Omega _2\cap dom L$*and*$\lambda \in (0,1)$*,*
(A6) $$\deg \{[I-(P+JQN)\gamma ]|_{\ker L}, \ker L\cap \Omega _2,0\}\ne 0$$*,*
(A7) *there exists*$u_0\in C\setminus \{0\}$*such that*$\Vert x\Vert \le \sigma (u_0)\Vert y\Vert$*for*$x\in C(u_0)\cap \partial \Omega _1$*and*$y\in \Psi x$*, where*$C(u_0)=\{x\in C:\mu u_0\preceq x ~for~some ~\mu >0 \}$*and*$\sigma (u_0)$*such that*$\Vert x+u_0\Vert \ge \sigma (u_0)\Vert x\Vert$*for every*$x\in C$*,*
(A8) $$(P+JQN)\gamma (\partial \Omega _2)\subset C$$*,*
(A9) $$\Psi _\gamma (\overline{\Omega }_2\setminus \Omega _1)\subset C$$*,*
(A10) $$x\notin (P+JQN)\gamma x ~for~x\in \partial \Omega _2\cap Ker L$$.

*Then the equation*$Lx\in Nx$*has at least one solution in the set*$C\cap (\overline{\Omega }_2\setminus \Omega _1)$.

## Main results

In this section, we state our result on the existence of positive solutions for (1).

For simplicity of notation, we set

$$G(t,s) ={\left\{ \begin{array}{ll} \alpha -\frac{\Gamma (\alpha +1)}{\Gamma (2\alpha )}{{(1-s)}^{\alpha }}- \frac{\alpha {{t}^{\alpha }}}{\Gamma (\alpha +1)}+ \frac{\alpha \Gamma (\alpha +1) {}}{\Gamma (2\alpha )},\quad 0\le t< s\le 1,\\ \alpha -\frac{\Gamma (\alpha +1)}{\Gamma (2\alpha )}{{(1-s)}^{\alpha }}- \frac{\alpha {{t}^{\alpha }}}{\Gamma (\alpha +1)}+ \frac{\alpha \Gamma (\alpha +1) {}}{\Gamma (2\alpha )}+\frac{1}{\Gamma (\alpha )}\left( \frac{t-s}{1-s}\right) ^{\alpha -1}, \quad 0\le s < t\le 1. \end{array}\right.}$$

By the monotonicity of the function, it is easy to verify that $G(t,s)>0$, $t,s\in [0,1]$. Here, we omit the proof. Moreover, $\kappa$ is a constant which satisfies

2 $$0<\kappa \le \min \left\{ 1,\;\frac{1}{\max _{t,s\in [0,1]}G(t,s)}\right\}.$$

Thus, we get $1-\kappa G(t,s)>0$, $t,s\in [0,1]$.

### **Theorem 2**

*Assume that:*

1. $f:[0,1]\times \mathbb {R}\rightarrow \mathcal {F}(\mathbb {R})$*,f(t,* *u)is continuous for every*$u\in \mathbb {R},~t\in [0,1]$*,*
2. *for each*$r>0$*, there exists*$\alpha _{r}\in L^1[0,1]$*such that*$|f(t,u)|\le \alpha _{r}(t)$*for a.e.*$t\in [0,1]$*and every*$u\in [0,r]$*, where*$|f(t,u)|=\sup \{|w|:w\in f(t,u)\}$*,*
3. *there exist positive constants*$b_1, b_2, b_3, c_1,c_2, B$*with*
  $$B>\frac{c_2}{c_1}+\frac{3b_2c_2}{\alpha b_1c_1}+\frac{{{3b}_{3}}}{\alpha {{b}_{1}}},$$
  *such that*
  $$-\kappa x\le w\le -c_1x+c_2 \quad and \quad w\le -b_1|w|+b_2x+b_3,$$
  *for all*$x\in [0,B]$*and*$w\in f(t,x)$*with*$t\in [0,1]$*,*
4. *there exist*$b\in (0,B)$*,*$t_0\in [0,1]$*,*$\rho \in (0,1]$*,*$\delta \in (0,1)$*and the function*$q\in L^1[0,1]$*,*$q(t)\ge 0, t\in [0,1]$*,*$h\in C\big ((0,b],\mathbb {R}^{+}\big )$*such that*$w(t,u)\ge q(t)h(u)$*for*$(t,u)\in [0,1] \times (0,b]$*and*$w\in f(t,u)$*.*$\frac{h(u)}{u^{\rho }}$*is non-increasing on (0, b] with*
  $$\frac{h(b)}{b}\int _0^1G(t_0,s)(1-s)^{\alpha -1}q(s)ds\ge \frac{1- \delta }{\delta ^{\rho }}.$$

*Then the problem* (1) *has at least one positive solution on [0, 1].*

### *Proof*

We use the Banach space $X=Y=C[0,1]$ with the supremum norm $\Vert x\Vert =\max _{t\in [0,1]}|x(t)|$.

Define $L:{\text{dom}}L \rightarrow X$ and $N:X \rightarrow 2^Y$ with ${\text{dom}}L=\left\{ x\in X:D_{0^+}^{\alpha }x(t)\in C[0,1], x^{(i)}(0)=0, x(0)=x(1),~i=1,2,\ldots ,n-1\right\}$ by

$$Lu=D_{0+}^{\alpha }u$$

and

$$Nu(t)=\left\{ y\in Y:y(t)\in f\left( t,u(t)\right) ~ \text {a.e. on}[0,1]\right\}.$$

Then the problem (1) can be written by

$$Lu\in Nu, \quad u\in {\text{dom}}L.$$

By Lemma 1, $D_{0^+}^{\alpha }u(t)=0$ has solution

$$u(t)=c_{0}+c_{1}t+\cdots +c_{n-1}t^{n-1},$$

where $c_i\in \mathbb {R}, i=0,1,\ldots ,n-1.$ According to the boundary conditions of (1), we get $c_i=0, i=1,2,\ldots ,n-1$. Thus, we obtain

$${\text{Ker}}L=\left\{ u\in {\text{dom}}L:u(t)=c\in \mathbb {R}\right\}.$$

Let $y\in {\text{Im}}L$, so there exists $u\in {\text{dom}}L$ which satisfies $Lu=y.$ By Lemma 1, we have

$$u(t)=I_{0+}^{\alpha }y(t)+{{c}_{0}}+{{c}_{1}}t+\cdots +{{c}_{n-1}}{{t}^{n-1}}.$$

By the definition of ${\text{dom}}L$, we have $c_i=0,~i=1,2,\ldots ,n-1$. Hence,

$$u(t)=I_{0+}^{\alpha }y(t)+c_{0}.$$

Taking into account $u(0)=u(1)$, we obtain

$$\int _{0}^{1}{{{(1-s)}^{\alpha -1}}y}(s)ds=0.$$

On the other hand, suppose *y* satisfies the above equation. Let $u(t)=I_{0+}^{\alpha }y(t)$, and we can easily prove $u(t)\in {\text{dom}} L$. Thus, we get

$${\text{Im}} L =\left\{ y\in Y:\int _{0}^{1}{{{(1-s)}^{\alpha -1}}y}(s)ds=0\right\}.$$

Define the linear continuous projector operator $P:X\rightarrow X$ by

$$Px(t)=\alpha \int _{0}^{1}{{{(1-s)}^{\alpha -1}}x}(s)ds,\quad t\in [0,1].$$

Next, we define the operator $Q:Y\rightarrow Y$ by

$$Qy(t)=\alpha \int _{0}^{1}{{{(1-s)}^{\alpha -1}}y}(s)ds,\quad t\in [0,1].$$

Noting that

$$\begin{aligned} P\left( Px(t)\right)&=\,\alpha \int _{0}^{1}{{{(1-s)}^{\alpha -1}}Px(t)}ds\\&=\,Px(t)\cdot \alpha \int _{0}^{1}{{{(1-s)}^{\alpha -1}}}ds\\&=\,Px(t), \end{aligned}$$

then we have $P^2=P$. Similarly, we have $Q^2=Q.$

Then, one has ${\text{Im}}P={\text{Ker}}L$ and ${\text{Ker}}Q={\text{Im}}L$. It follows from ${\text{Ind}}L = {\text{dim}} ({\text{ker}}L) -{\text{codim}} ({\text{Im}}L) =0$ that *L* is a Fredholm mapping of index zero. Then, (A1) holds.

We consider the mapping $K_P:{\text {Im}}L\rightarrow {\text {dom}}L \cap {\text{Ker}} P$ by

$$K_Py(t)=\int _{0}^{1}k(t,s)y(s)ds,\quad t\in [0,1],$$

where

$$k(t,s):={\left\{ \begin{array}{ll} \frac{1}{\Gamma ( \alpha )}(t-s)^{\alpha -1} - \frac{\Gamma (1+\alpha )}{\Gamma (2\alpha )}{(1-s)}^{2\alpha -1},\quad 0\le s\le t\le 1,\\ -\frac{\Gamma (1+\alpha )}{\Gamma (2\alpha )}{(1-s)}^{2\alpha -1}, \quad 0\le t< s\le 1, \end{array}\right. }$$

Now, we will prove that is $K_P$ the inverse of $L|_{\text{dom}}L\cap {\text{Ker}}P$. In fact, for $x\in {\text{dom}}L\cap {\text{Ker}}P$, we have $D_{0+}^{\alpha }x(t)=y(t)\in {\text{Im}}L$ and $\alpha \int _{0}^{1}{{{(1-s)}^{\alpha -1}}x}(s)ds=0.$

By Lemma 1, one has

$$(K_Py)(t)=x(t)=I_{0+}^{\alpha }y(t)+c_0+c_1t+\cdots +c_{n-1}t^{n-1}.$$

According to the definition of ${\text{dom}}L$, we get $c_i=0,i=1,2,\ldots ,n-1$. Furthermore, by $\alpha \int _{0}^{1}{{{(1-s)}^{\alpha -1}}x}(s)ds=0,$ we have $c_0=-\Gamma (1+\alpha )(I_{0+}^{2\alpha }y)(1).$

Thus,

$$\begin{aligned} (K_Py)(t)&=\,I_{0+}^{\alpha }y(t)+c_0=I_{0+}^{\alpha }y(t)-\Gamma (1+\alpha )\left( I_{0+}^{2\alpha }y\right) (1)\\&=\,I_{0+}^{\alpha }y(t)-\Gamma (1+\alpha )\cdot \frac{1}{\Gamma (2\alpha )}\int _{0}^{1}{{{(1-s)}^{2\alpha -1}}y}(s)ds\\&=\,\int _{0}^{1}k(t,s)y(s)ds. \end{aligned}$$

Obviously, $LK_Py=y$. Moreover, for $x\in {\text{dom}}L\cap {\text{Ker}}P$, we get $\alpha \int _{0}^{1}{{{(1-s)}^{\alpha -1}}x}(s)ds=0$ and

$$\begin{aligned} K_PLx&=\,I_{0+}^{\alpha }D_{0+}^{\alpha }x(t)-\Gamma (1+\alpha )\left( I_{0+}^{2\alpha }D_{0+}^{\alpha }x\right) (1) \\&=\,x(t)-x(0)-\Gamma (1+\alpha )\left( I_{0+}^{\alpha }I_{0+}^{\alpha }D_{0+}^{\alpha }x\right) (1)\\&=\,x(t)-x(0)-\Gamma (1+\alpha )I_{0+}^{\alpha }\left( x(t)-x(0)\right) |_{t=1}\\&=\,x(t)-x(0)-\Gamma (1+\alpha )I_{0+}^{\alpha }x(1)+\Gamma (1+\alpha )I_{0+}^{\alpha }(x(0))|_{t=1}\\&=\,x(t)-x(0)-\frac{\Gamma (1+\alpha )}{\Gamma (\alpha )}\int _0^1(1-s)^{\alpha -1}x(s)ds +\frac{\Gamma (1+\alpha )}{\Gamma (\alpha )}\int _0^1(1-s)^{\alpha -1}x(0)ds\\&=\,x(t)-x(0)-\alpha \int _{0}^{1}{{{(1-s)}^{\alpha -1}}x}(s)ds+x(0)\\&=\,x(t). \end{aligned}$$

Thus, we know that $K_P=\left( L|_{\text{dom}}L\cap {\text{Ker}}P\right) ^{-1}$. Moreover, it is easy to see that

3 $$|k(t,s)|\le 3(1-s)^{\alpha -1},\quad \forall t,s\in [0,1].$$

Consider the cone

$$C=\left\{ x\in X: x(t)\ge 0, ~t\in [0,1]\right\}.$$

It is clear that (H1) and (H2) imply (A2) and (A3).

Let

$$\begin{aligned} \Omega _1= & {} \left\{ x\in X: \delta \Vert x\Vert<|x(t)|<b,~t\in [0,1]\right\} ,\\ \Omega _2= & {} \left\{ x\in X:\Vert x\Vert <B\right\} . \end{aligned}$$

Clearly, $\Omega _1$ and $\Omega _2$ are bounded and open sets and

$$\overline{\Omega }_1=\left\{ x\in X: \delta \Vert x\Vert \le |x(t)|\le b,~t\in [0,1]\right\} \subset \Omega _2.$$

Moreover, $C\cap (\overline{\Omega }_2\setminus \Omega _1)\ne \emptyset$. Let $J=I$ and $(\gamma x)(t)=|x(t)|$ for $x\in X$, then $\gamma$ is a retraction and maps subsets of $\overline{\Omega }_2$ into bounded subsets of *C*, which means that (A4) holds.

Next, we will show (A5) holds. Suppose that there exist $u_0\in \partial \Omega _2\cap C\cap {\text {dom}}L$ and $\lambda _0\in (0,1)$ such that $Lu_0\in \lambda _0Nu_0$, then $D_{0^+}^{\alpha }u_0(t)\in \lambda _0f(t,u_0(t))$ for all $t\in [0,1]$. In view of (H3), we get that there exists $w^*\in f(t,u_0(t))$ such that

4 $$\begin{aligned} D_{0^+}^{\alpha }u_0(t)&=\,\lambda _0 w^* \le -\lambda _0b_1|w^*|+\lambda _0b_2u_0(t)+\lambda _0b_3 \\&=\,-b_1\left| D_{0^+}^{\alpha }u_0(t)\right| +\lambda _0b_2u_0(t)+\lambda _0b_3 \\&\le \, -b_1\left| D_{0^+}^{\alpha }u_0(t)\right| +b_2u_0(t)+b_3, \end{aligned}$$

and

5 $$D_{0^+}^{\alpha }u_0(t)=\lambda _0 w^* \le -\lambda _0c_1u_0(t)+\lambda _0c_2,$$

From (4), we obtain

$$\begin{aligned} 0&=u_0(0)-u_0(1)=\left( I_{0+}^{\alpha }D_{0+}^{\alpha }u_0\right) (1)\\&\le \, -\frac{{{b}_{1}}}{\Gamma (\alpha )}\int _{0}^{1}{{{(1-s)}^{\alpha -1}}\left| D_{0+}^{\alpha }{{u}_{0}}(s) \right| ds+}\frac{{{b}_{2}}}{\Gamma (\alpha )}\int _{0}^{1}{{{(1-s)}^{\alpha -1}}{{u}_{0}}(s)ds}\\ {}&\quad +\,\frac{{{b}_{3}}}{\Gamma (\alpha )}\int _{0}^{1}{{{(1-s)}^{\alpha -1}}ds}, \end{aligned}$$

which gives

$$\int _{0}^{1}{{{(1-s)}^{\alpha -1}}\left| D_{0+}^{\alpha }{{u}_{0}}(s) \right| ds}\le \frac{{{b}_{2}}}{{{b}_{1}}}\int _{0}^{1}{{{(1-s)}^{\alpha -1}}{{u}_{0}}(s)ds+}\frac{{{b}_{3}}}{\alpha {{b}_{1}}}.$$

From (5), we obtain

$$\int _{0}^{1}{{{(1-s)}^{\alpha -1}}{{u}_{0}}(s)ds\le }\frac{{{c}_{2}}}{\alpha {{c}_{1}}}.$$

From (3) and the equation

$${{u}_0}=(I-P){{u}_0}+P{{u}_0}={{K}_{P}}L(I-P){{u}_0}+ P{{u}_0}=P{{u}_0}+{{K}_{P}}L{{u}_0},$$

we can get

$$\begin{aligned} {u}_{0}&=\,\alpha \int _{0}^{1}{{{(1-s)}^{\alpha -1}}{u}_{0}}(s)ds+\int _{0}^{1}k(t,s)D_{0+}^{\alpha }u_0(s)ds\\&\le \,\frac{c_2}{c_1}+\int _{0}^{1}|k(t,s)|\cdot \left| D_{0+}^{\alpha }u_0(s)\right| ds\\&=\,\frac{c_2}{c_1}+\int _{0}^{1}\frac{|k(t,s)|}{(1-s)^{\alpha -1}}\cdot (1-s)^{\alpha -1}\left| D_{0+}^{\alpha }u_0(s)\right| ds\\&\le \,\frac{c_2}{c_1}+3\int _{0}^{1}(1-s)^{\alpha -1}\left| D_{0+}^{\alpha }u_0(s)\right| ds\\&\le \, \frac{c_2}{c_1}+3\left[ \frac{{{b}_{2}}}{{{b}_{1}}}\int _{0}^{1}{{{(1-s)}^{\alpha -1}}{{u}_{0}}(s)ds+}\frac{{{b}_{3}}}{\alpha {{b}_{1}}} \right] \\&\le \,\frac{c_2}{c_1}+\frac{3b_2c_2}{\alpha b_1c_1}+\frac{{{3b}_{3}}}{\alpha {{b}_{1}}}. \end{aligned}$$

Then, we have

$$B=\Vert u_0\Vert \le \frac{c_2}{c_1}+\frac{3b_2c_2}{\alpha b_1c_1}+\frac{{{3b}_{3}}}{\alpha {{b}_{1}}},$$

which contradicts (H3). Hence (A5) holds.

To prove (A6), consider $x\in \text {Ker}L\cap \overline{\Omega }_2$, then $x(t)\equiv c$ on [0, 1]. Let

$$\begin{aligned} H(c,\lambda )&=\,[I-\lambda (P+JQN)\gamma ]c\\&=\,c-\lambda \alpha \int _{0}^{1}{{(1-s)}^{\alpha -1}}|c|ds-\lambda \alpha \int _0^1{{(1-s)}^{\alpha -1}} f(s,|c|)ds\\&=\,c-\lambda |c|-\lambda \alpha \int _0^1{{(1-s)}^{\alpha -1}} f(s,|c|)ds, \end{aligned}$$

for $c\in [-B,B]$ and $\lambda \in [0,1]$. It is easy to show that $0\in H(c,\lambda )$ implies $c\ge 0$. Suppose $0\in H(B,\lambda )$ for some $\lambda \in (0,1]$. Then,

$$0=B-\lambda B-\lambda \alpha \int _0^1{{(1-s)}^{\alpha -1}}w(s,B)ds,$$

where $w\in f(t,B),~t\in [0,1]$. So (H3) leads to

$$0\le B(1-\lambda )=\lambda \alpha \int _0^1{{(1-s)}^{\alpha -1}}w(s,B)ds\le \lambda (-c_1B+c_2)<0,$$

which is a contradiction. In addition, if $\lambda =0$, then $B=0$, which is impossible. Thus, $H(x,\lambda )\ne 0$ for $x\in \text {Ker} L\cap \partial {\Omega }_2$, $\lambda \in [0,1]$. As a result,

$$\begin{aligned} \deg \{&[I-(P+\,JQN)\gamma ]_{\text {Ker} L}, \text {Ker} L\cap \Omega _2,0\} \\&=\deg \{H(\cdot ,1), \text {Ker} L\cap \Omega _2,0\}\\&=\deg \{H(\cdot ,0), \text {Ker} L\cap \Omega _2,0\}\\&=\deg \{I, \text {Ker} L\cap \Omega _2,0\}=1 \ne 0. \end{aligned}$$

So (A6) holds.

Next, we prove (A7). Letting $u_0(t)\equiv 1$, so we have $u_0\in C\setminus \{0\}$ and $C(u_0)=\{x\in C:x(t)>0, t\in [0,1]\}$. We can take $\sigma (u_0)=1$. For $x\in C(u_0)\cap \partial \Omega _1$, we get $x(t)>0$, $0<\Vert x\Vert \le b$ and $x(t)\ge \delta \Vert x\Vert ,~t\in [0,1]$.

By (H3) and (H4), for every $x\in C(u_0)\cap \partial \Omega _1$ and $v\in \Psi x$, there exits $w\in Nx$ such that

$$\begin{aligned} v(t_0)&=\,\alpha \int ^1_0(1-s)^{\alpha -1}x(s)ds+ \int ^1_0G(t_0,s)(1-s)^{\alpha -1}w\left( s,x(s)\right) ds\\&\ge \, \delta \Vert x\Vert +\int _0^1G(t_0,s)(1-s)^{\alpha -1}q(s)h\left( x(s)\right) ds\\&=\,\delta \Vert x\Vert +\int _0^1G(t_0,s)(1-s)^{\alpha -1}q(s)\cdot \frac{h\left( x(s)\right) }{x^{\rho }(s)} x^{\rho }(s)ds\\&\ge \,\delta \Vert x\Vert +\delta ^{\rho }\Vert x\Vert ^{\rho }\int _0^1G(t_0,s)(1-s)^{\alpha -1} q(s)\cdot \frac{h(b)}{b^{\rho }}ds\\&=\, \delta \Vert x\Vert +\delta ^{\rho }\Vert x\Vert \cdot \frac{b^{1-\rho }}{\Vert x\Vert ^{1-\rho }}\int _0^1G(t_0,s)(1-s)^{\alpha -1}q(s) \frac{h(b)}{b}ds\\&\ge \, \delta \Vert x\Vert +\delta ^{\rho }\Vert x\Vert \cdot \int _0^1G(t_0,s)(1-s)^{\alpha -1}q(s) \frac{h(b)}{b}ds\\&\ge \,\delta \Vert x\Vert +\delta ^{\rho }\Vert x\Vert \cdot \frac{1-\delta }{\delta ^\rho }\\&=\Vert x\Vert . \end{aligned}$$

Thus, $\Vert x\Vert \le \sigma (u_0)\Vert \Psi x\Vert$ for all $x\in C(u_0)\cap \partial \Omega _1$, i.e., (A7) holds.

Since for $x\in \partial \Omega _2$ and $w\in N\gamma x$, from (H2) we have

$$\begin{aligned} (P+JQN)\gamma x&=\, \alpha \int _{0}^{1}{{{(1-s)}^{\alpha -1}}|x(s)|}ds+ \alpha \int _{0}^{1}{{{(1-s)}^{\alpha -1}}}w(s,|x(s)|)ds\\&\ge \, \alpha \int _0^1(1-s)^{\alpha -1}(1-\kappa )|x(s)|ds\\&\ge \,0. \end{aligned}$$

Thus, $(P+JQN)\gamma x\subset C$ for $x\in \partial \Omega _2$. Then (A8) holds.

Next, we prove (A9). Let $x\in \overline{\Omega }_2{\setminus} \Omega _1$

$$\begin{aligned} \Psi _\gamma x(t)&=\,\left\{ v\in X: \exists w\in N_\gamma x~ \text {such that} ~v=\alpha \int ^1_0(1-s)^{\alpha -1}|x|ds\right. \\&\left. \qquad +\,\int ^1_0G(t,s){(1-s)^{\alpha -1}}w(s,|x|)ds\right\} . \end{aligned}$$

According to (H3) and (2), for $x\in \overline{\Omega }_2{\setminus }\Omega _1$ and $v\in \Psi _\gamma x$, there exits $w\in N_\gamma x$ such that

$$\begin{aligned} v(t)&=\,\alpha \int ^1_0(1-s)^{\alpha -1}|x(s)|ds+ \int ^1_0G(t,s){(1-s)^{\alpha -1}}w\left( s,|x(s)|\right) ds\\&>\,\int ^1_0(1-s)^{\alpha -1}|x(s)|\left( 1-\kappa G(t,s)\right) ds\\&\ge \, 0. \end{aligned}$$

Hence, $$\Psi _\gamma \left( \overline{\Omega }_2{\setminus }\Omega _1\right) \subset C$$; i.e., (A9) holds.

To prove (A10), suppose there exists $u_0\in \partial \Omega _2\cap \text {Ker}L$, i.e., $u_0=c\in \mathbb {R}$ and $|c|=B$ such that $c\in (P+JQN)\gamma u.$ For $w\in N\gamma c$, we have

$$\begin{aligned} c&=\,\alpha \int ^1_0(1-s)^{\alpha -1}|c|ds+ \alpha \int ^1_0(1-s)^{\alpha -1}w(s,|c|)ds\\&\ge \,\alpha \int _0^1(1-s)^{\alpha -1}(1-\kappa )|c|ds\\&\ge \,0. \end{aligned}$$

Hence, we get $c\in (P+JQN)\gamma u$ implies $c\ge 0$. Then for $c=B$ and $w\in N\gamma B$, we have

$$B=\alpha \int ^1_0(1-s)^{\alpha -1}Bds+\alpha \int ^1_0(1-s)^{\alpha -1}w(s,B)ds.$$

Hence,

$$\alpha \int ^1_0(1-s)^{\alpha -1}w(s,B)ds=0.$$

On the other hand, from (H3), we have

$$0=\alpha \int ^1_0(1-s)^{\alpha -1}w(s,B)ds\le -c_1B+c_2<0.$$

This contradiction implies (A10) holds.

Hence, applying Theorem 1, BVP (1) has a positive solution $u^{*}$ on [0, 1] with $b\le \Vert u^{*}\Vert \le B$. This completes the proof. $\square$

## Example

To illustrate how our main result can be used in practice, we present here an example.

Let us consider the following fractional differential inclusion at resonance

6 $${\left\{ \begin{array}{ll} D_{0^+}^{1.5}u(t)\in f(t,u),\quad 0\le t\le 1, \\ u^{\prime }(0)=0,\quad u(0)=u(1), \end{array}\right. }$$

where $$f(t,u)=\left\{ w(t,u)+\frac{1}{25}v:v\in [0,1]\right\}$$, $$w(t,u)=\frac{1}{300}\left( 1+2t-2t^2\right) \left( u^{2}-4u+3\right) u.$$

Corresponding to BVP (1), we have that $\alpha =1.5$ and

$$G(t,s) ={\left\{ \begin{array}{ll} \frac{3}{2} -\frac{\Gamma (2.5)}{\Gamma (3)}{{(1-s)}^{1.5 }}- \frac{1.5 {{t}^{1.5 }}}{\Gamma (2.5)}+ \frac{1.5\Gamma (2.5)}{\Gamma (3)},\quad 0\le t< s\le 1,\\ \frac{3}{2} -\frac{\Gamma (2.5)}{\Gamma (3)}{{(1-s)}^{1.5 }}- \frac{1.5 {{t}^{1.5 }}}{\Gamma (2.5)}+ \frac{1.5\Gamma (2.5)}{\Gamma (3)}+\frac{1}{\Gamma (1.5)}\left( \frac{t-s}{1-s}\right) ^{0.5},\quad 0\le s < t\le 1, \end{array}\right. }$$

It is easy to see that $G(t,s)\ge 0$ for $t,s\in [0,1]$.

Let $\kappa =0.003$ and $B=2$. By the monotonicity of the function, for $x\in [0,2]$ and $w\in f(t,x)$, $t\in [0,1]$, we can prove that

$$-\frac{3}{1000} x\le w(t,x)\le -\frac{1}{30}x+\frac{1}{17}$$

and

$$w(t,x)\le -\frac{8}{3}|w|+\frac{1}{30}x+\frac{1}{4}.$$

Then, we can choose $c_1=\frac{1}{30}$, $c_2=\frac{1}{17}$, $b_1=\frac{8}{3}$, $b_2=\frac{1}{30}$, $b_3=\frac{1}{4}$. By calculation, we have

$$\frac{c_2}{c_1}+\frac{3b_2c_2}{\alpha b_1c_1}+\frac{{{3b}_{3}}}{\alpha {{b}_{1}}}\approx 1.764+0.044+0.187=1.808<2=B.$$

Take $q(t)=\frac{1}{240}\left( 1+2t-t^2\right)$ and $h(x)=x$. We see that $q\in L^1[0,1]$, $q(t)\ge 0$ and $h\in C\big ((0,b],\mathbb {R}^{+}\big ),$ where $b=1/2\in (0,B)=(0,2)$. Furthermore, for $(t,u)\in [0,1] \times (0,1/2]$ and $w\in f(t,u)$, by a simple computation, we get that

$$w(t,u)\ge q(t)h(u).$$

Choose $\rho =1$, so we have $\frac{h(u)}{u^{\rho }}\equiv 1$ which is non-increasing on (0, *b*]. By Choosing $t_0=0$, $\delta =0.997$, with simple calculations, we can get

$$\frac{h(b)}{b}\int _0^1G(t_0,s)(1-s)^{\alpha -1}q(s)ds\ge \frac{1- \delta }{\delta ^{\rho }}.$$

Therefore, (H$_1$)–(H$_4$) of Theorem 2 are satisfied. Then BVP (6) has a positive solution on [0, 1].

## Conclusions

In this paper, we have obtained the existence of positive solutions for a boundary value problem of fractional differential inclusions at resonance. By using the Leggett–Williams theorem for coincidences of multi-valued operators due to O’Regan and Zima, we have found the existence results. Our results are new in the context of fractional differential inclusions and positive solutions. As applications, an example is presented to illustrate the main results. In the future, we will consider the the uniqueness of positive solutions for the fractional differential equations at resonance.

## References

[CR1] Agarwal RP, Benchohra M, Hamani S (2010). A survey on existence results for boundary value problems of nonlinear fractional differential equations and inclusions. Acta Appl Math.

[CR2] Bai Z (2011). Solvability for a class of fractional m-point boundary value problem at resonance. Comput Math Appl.

[CR3] Bai Z, Lü H (2005). Positive solutions for boundary value problem of nonlinear fractional differential equation. J Math Anal Appl.

[CR4] Bai Z, Zhang Y (2011). Solvability of fractional three-point boundary value problems with nonlinear growth. Appl Math Comput.

[CR5] Caballero J, Harjani J, Sadarangani K (2011). Positive solutions for a class of singular fractional boundary value problems. Comput Math Appl.

[CR6] Chen Y, Tang X, He X (2013). Positive solutions of fractional differential inclusion at resonace. Mediterr J Math.

[CR7] Goodrich CS (2010). Existence of a positive solution to a class of fractional differential equation. Appl Math Lett.

[CR8] Kilbas AA, Srivastava HM, Trujillo JJ (2006). Theory and applications of fractional differential equations. North-Holland mathematics studies.

[CR9] Kosmatov N (2008). Multi-point boundary value problems on an unbounded domain at resonance. Nonlinear Anal.

[CR10] Kosmatov N (2010). A boundary value problem of fractional order at resonance. Electron J Differ Equ.

[CR11] Lin W (2007). Global existence theory and chaos control of fractional differential equations. J Math Anal Appl.

[CR12] Mawhin J (1979). Topological degree methods in nonlinear boundary value problems. NSFCBMS regional conference series in mathematics.

[CR13] O’Regan D, Zima M (2008). Leggett–Williams theorems for coincidences of multivalued operators. Nonlinear Anal.

[CR14] Orsingher E, Beghin L (2004). Time-fractional telegraph equations and telegraph processes with brownian time. Probab Theory Relat Fields.

[CR15] Podlubny I (1999). Fractional differential equations.

[CR16] Samko SG, Kilbas AA, Marichev OI (1993). Fractional integrals and derivatives. Theory and applications.

[CR17] Xu X, Jiang D, Yuan C (2009). Multiple positive solutions for the boundary value problem of a nonlinear fractional differential equation. Nonlinear Anal.

[CR18] Yang A, Wang H (2011). Positive solutions for two-point boundary value problems of nonlinear fractional differential equation at resonace. Electron J Qual Theory Differ Equ.

[CR19] Zhang S (2006). Positive solutions for boundary-value problems of nonlinear fractional differential equations. Electron J Differ Equ.

